# Maize *LOST SUBSIDIARY CELL* encoding a large subunit of ribonucleotide reductase is required for subsidiary cell development and plant growth

**DOI:** 10.1093/jxb/erad153

**Published:** 2023-04-27

**Authors:** Yongqi Cui, Meiqing He, Jie Liu, Shuang Wang, Junli Zhang, Shiyi Xie, Zhubing Hu, Siyi Guo, Dawei Yan

**Affiliations:** State Key Laboratory of Crop Stress Adaptation and Improvement, School of Life Sciences, Academy for Advanced Interdisciplinary Studies, Henan University, Kaifeng 475004, China; State Key Laboratory of Crop Stress Adaptation and Improvement, School of Life Sciences, Academy for Advanced Interdisciplinary Studies, Henan University, Kaifeng 475004, China; State Key Laboratory of Crop Stress Adaptation and Improvement, School of Life Sciences, Academy for Advanced Interdisciplinary Studies, Henan University, Kaifeng 475004, China; State Key Laboratory of Crop Stress Adaptation and Improvement, School of Life Sciences, Academy for Advanced Interdisciplinary Studies, Henan University, Kaifeng 475004, China; State Key Laboratory of Crop Stress Adaptation and Improvement, School of Life Sciences, Academy for Advanced Interdisciplinary Studies, Henan University, Kaifeng 475004, China; Maize Engineering and Technology Research Center of Hunan Province, College of Agronomy, Hunan Agricultural University, Changsha 410128, China; State Key Laboratory of Crop Stress Adaptation and Improvement, School of Life Sciences, Academy for Advanced Interdisciplinary Studies, Henan University, Kaifeng 475004, China; State Key Laboratory of Crop Stress Adaptation and Improvement, School of Life Sciences, Academy for Advanced Interdisciplinary Studies, Henan University, Kaifeng 475004, China; State Key Laboratory of Crop Stress Adaptation and Improvement, School of Life Sciences, Academy for Advanced Interdisciplinary Studies, Henan University, Kaifeng 475004, China; Cardiff University, UK

**Keywords:** dNTP, growth, maize, ribonucleotide reductase, stomata, subsidiary cell

## Abstract

The four-celled stomatal complex consists of a pair of guard cells (GCs) and two subsidiary cells (SCs) in grasses, which supports a fast adjustment of stomatal aperture. The formation and development of SCs are thus important for stomatal functionality. Here, we report a maize *lost subsidiary cells* (*lsc*) mutant, with many stomata lacking one or two SCs. The loss of SCs is supposed to have resulted from impeded subsidiary mother cell (SMC) polarization and asymmetrical division. Besides the defect in SCs, the *lsc* mutant also displays a dwarf morphology and pale and striped newly-grown leaves. *LSC* encodes a large subunit of ribonucleotide reductase (RNR), an enzyme involved in deoxyribonucleotides (dNTPs) synthesis. Consistently, the concentration of dNTPs and expression of genes involved in DNA replication, cell cycle progression, and SC development were significantly reduced in the *lsc* mutant compared with the wild-type B73 inbred line. Conversely, overexpression of maize *LSC* increased dNTP synthesis and promoted plant growth in both maize and Arabidopsis. Our data indicate that LSC regulates dNTP production and is required for SMC polarization, SC differentiation, and growth of maize.

## Teaser

The maize *LSC*gene, encoding a large subunit of ribonucleotide reductase, modulates dNTP synthesis, subsidiary cell development and plant growth.

## Introduction

Stomata are important pores for gas and water vapour exchange with the environment, which are located on the outermost cellular layer of leaves, stems, and other parts of plants, (Hetherington and Woodward, 2003). In addition, they also allow pathogens, including fungi, bacteria, and nematodes, to gain access into the plant (Schroeder *et al.*, 2001; Melotto *et al.*, 2006). To balance the entry and exit of molecules delicately, or respond to the exterior stimuli in time, stomata must be able to open and close very quickly. This opening and closure are mediated by rapid changes in the turgor pressure of the two guard cells (GCs), which is controlled by movements of ions and sugars into and out of GCs via membrane transporters (Schroeder *et al.*, 2001; Pandey *et al.*, 2007; Kim *et al.*, 2010; Engineer *et al.*, 2016). Thus, mature GCs lose symplastic connections with neighbouring cells to keep relatively isolated, and facilitate the fine control of volume changes (Wille and Lucas, 1984; Palevitz and Hepler, 1985).

The development of the stomatal complex is coordinated by a variety of factors, including transcription factors, phosphokinase pathways, peptide receptors and ligands, and endogenous hormones (Hara *et al.*, 2009; MacAlister and Bergmann, 2011; Chater *et al.*, 2016; Wang *et al.*, 2019; Wu *et al.*, 2019). Meanwhile, exogenous signals such as CO_2_, light, moisture, and temperature also play indispensable roles (Gray *et al.*, 2000; Thomas *et al.*, 2004; Coupe *et al.*, 2006; Engineer *et al.*, 2014; Wang *et al.*, 2021). The stomatal complexes vary from monocots to dicots not only in the composition, but also in the developmental process. Stomata in dicots such as Arabidopsis are relatively simple, consisting of two kidney-shaped GCs. In contrast, stomata of monocots, such as rice and maize, contain two dumbbell-shaped GCs flanked by a pair of subsidiary cells (SCs). The developmental process of monocot stomata can be generally divided into the following stages: (i) the process is initiated by SPEECHLESS (SPCH) and SCREAM (SCRM)/SCRM 2 in protodermal cells, which are transformed into guard mother cells (GMCs) through continuous asymmetric division; (ii) a heterodimer composed of MUTE and SCRM/SCRM 2 promotes the formation of subsidiary mother cells (SMCs) that undergo asymmetric division to generate SCs; (iii) GCs are formed by a symmetrical division of GMCs, which is directed by the transcription factors FAMA and SCRM/SCRM 2 (Ohashi-Ito and Bergmann, 2006; Pillitteri *et al.*, 2007; Kanaoka *et al.*, 2008; Liu *et al.*, 2009; Simmons and Bergmann, 2016). Two homologous MYB transcription factors, MYB88 and FLP (FOUR LIPS), have also been shown to be involved in this process (Lee *et al.*, 2013). CYCLIN DEPENDENT KINASE B1s (CDKB1s) and CYCLIN A2s (CYCA2s) are downstream target proteins for FLP and MYB88 (Yang *et al.*, 2014). They synergistically inhibit the differentiation of GMC. Among the regulators of monocot stomatal development, a notable transcription factor is the mobile BZU2/ZmMUTE, which moves from the GMC to SMCs, and transcriptionally regulates a number of genes implicated in SC development. The *bzu2-1* mutant showed defects in both GMC and GC division and differentiation (Wang *et al.*, 2019).

During asymmetric division, the polarization of SMCs determines SC production. Two leucine-rich receptor-like proteins PANGLOSS (PAN)1/2, expressed in SMCs, regulate this asymmetric division of SMCs (Cartwright *et al.*, 2009; Zhang *et al.*, 2012). PAN1 interacts with Rho family GTPase (ROP) 2/9, promoting the formation of actin plates. The nuclei of SMCs are then pulled to the side close to GMCs, thereby motivating the asymmetric division of SMC (Jeon *et al.*, 2008; Humphries *et al.*, 2011). In addition, BRICK3 (BRK3), a member of the SCAR/WAVE family, can also promote asymmetric division of SMC (Facette *et al.*, 2015). At present, the function of SCs is known to only protect GCs and assist their movement (DeMichele and Sharpe, 1973; Edwards *et al.*, 1976). However, the underlying regulatory mechanisms of their development and function are not well understood.

DNA, acting as the genetic material of organisms, is synthesized by DNA polymerase using dNTPs as raw materials. To ensure high-fidelity DNA replication and genomic stability, intracellular dNTPs concentrations must be maintained within a relatively reasonable range. The change of dNTP content also affects many aspects of plant growth and development, such as DNA repair, cell division, and chloroplast development (Yoo *et al.*, 2009; Schaaper and Mathews, 2013; Niu *et al.*, 2017; Xie *et al.*, 2020). Ribonucleotide reductase (RNR) catalyses the reduction of nucleoside diphosphates (NDPs) to deoxy-ribonucleoside diphosphates (dNDPs), the most important rate-limiting step in the *de novo* synthesis of dNTPs (Kolberg *et al.*, 2004; Witte and Herde, 2020; Straube *et al.*, 2021). Therefore, the fine regulation of RNR enzyme activity is essential for maintaining dNTP balance and genome stability *in vivo*. RNR functions as a heterologous tetramer, composed by two large (RNRL) and two small (RNRS) subunits (Elledge *et al.*, 1992; Jordan and Reichard, 1998). The RNRL subunit binds to the nucleoside diphosphate (NDP) substrates and allosteric effectors, and is the target of feedback regulation, which ensures that dNTPs are not overproduced, and that enough NDPs are left for RNA synthesis. The RNRS subunit houses the di-iron tyrosyl radical co-factor indispensable for the reduction of NDPs to dNDPs (Elledge *et al.*, 1992; Stubbe, 1998, 2003). In Arabidopsis, *TSO2*, *RNR2A*, and *RNR2B*, encoding the RNRSs, show functional redundancy in modulating cell cycle progression and plant development (Wang and Liu, 2006). *Crinkled Leaves 8 (CLS8/RNR1)* encodes the Arabidopsis RNRL, while the *cls8-1* mutant has fewer copies of the chloroplast genome, crinkled leaves, bleached leaf sectors, and fewer but larger chloroplasts than wild-type plants (Garton *et al.*, 2007). *Virescent3* (*V3*) and *Stripe1* (*St1*), encoding *OsRNRL1* and *OsRNRS1*, respectively, in rice function in the process of plastid transcription and translation (Yoo *et al.*, 2009). *ZmRNRL1*, encodes the RNRL in maize; a nucleotide replacement of G-to-A in *Thermosensitive vanishing tassel1-R (Tvt1-R)* caused an amino acid substitution of Arg277-to-His277 in ZmRNRL1, and could not produce tassels at high temperatures (Xie *et al.*, 2020). Overall, RNRs have proved to be critical for cell division, DNA damage repair, chloroplast biogenesis, as well as plant growth and development.

Here, we identify maize *LOST SUBSIDIARY CELL (LSC)* gene, encoding a large subunit of RNR. The loss-of-function *lsc-1* and *lsc-2* maize mutants display aberrant stomata with one or two SC depletions, and compromised plant growth. In contrast, *LSC* overexpression promotes the vegetative growth in both maize and Arabidopsis. Furthermore, the *lsc* mutation disturbs dNTP biosynthesis and regulates the expression of several essential genes involved in cell cycle and stomata development. Our findings suggest that *LSC* attributes to *in vivo* dNTP synthesis, and is required for normal SC development and plant growth in maize.

## Materials and methods

### Plant materials and growth conditions

The *lsc-1* and *lsc-2* maize mutants were obtained from an ethyl methanesulfonate-mutagenized mutant library (Maize EMS-induced Mutant Database; http://www.elabcaas.cn/memd/; Lu *et al.*, 2018) in the wild type B73 genetic background. Maize plants were grown in the field in Henan University, Kaifeng, Henan Province, and Sanya, Hainan Province, China. The transgenic maize plants were grown in a greenhouse under 16 h light/8 h dark at 28–30 °C. *Arabidopsis thaliana* Columbia-0 (Col-0) plants were grown in a plant incubator under 16 h light (120 μmol m^–2^ s^–1^)/8 h dark cycle at 22 °C and 65% relative humidity.

### Stomatal density and stomatal aperture

The lower epidermis of second leaves from 8-day-old maize seedlings was carefully scraped with scalpel. The number of stomata was counted using a Zeiss Axioskop II microscope (Zeiss, Germany) equipped with differential interference contrast optics. The number of stomata per mm^2^ was represented as stomatal density. Stomatal aperture was measured in abaxial epidermal peels from fully expanded leaves (Yoo *et al*., 2010). The epidermis was incubated in buffer solution (20 mM KCl, 5 mM MES-KOH, and 1 mM CaCl_2_, pH 6.15) for 4 h, and then placed onto a slide, and stomatal apertures were measured using a Zeiss LSM710 confocal microscope under bright field.

### Light, fluorescence, and confocal microscopy

To determine the stomata phenotype, epidermal strips were peeled from mature leaves of wild type, *lsc-1*, and *lsc-2* maize plants, and were observed using a Zeiss Axioskop II microscope equipped with differential interference contrast optics. To observe the process of stomatal complex development, ~1.5 cm of segments from the leaf base were excised from 8-day-old seedlings and examined.

For F-actin analysis, 1.5 cm of basal leaf segments were excised from 8-day-old seedlings and cut into 0.2 cm wide and 0.5 cm long strips, followed by fixation in a solution containing 4% paraformaldehyde dissolved in 50 mM PEM (50 mM PIPES, 2.5 mM EGTA, 2.5 mM MgCl_2_) for 30 min at 25 °C (Wang *et al.*, 2019). The strips were washed three times for 5 min in 50 mM PEM, and were permeabilized by submersion for 20 min in 50 mM PEM containing 5% DMSO and 1% Triton X-100. After three more washes in 50 mM PEM, the sections were incubated at 25 °C for 1.5 h in a 50 mM PEM solution containing 90 nM AlexaFluor 488-phalloidin (dissolved in DMSO, Invitrogen, USA). Images were captured using a Zeiss LSM710 confocal microscope. Alexa Fluor 488 was detected at 488 nm excitation/500–560 nm emission.

To examine SCs, the epidermal peels from wild type, *lsc-1*, and *lsc-2* mutant maize leaves were stained with a solution of 0.1% toluidine blue for 1–2 min, and then washed with distilled or deionized water gently for 3–5 min, followed by drying before microscopic examination.

To identify the positive transgenic overexpression plants, the VENUS fluorescence of 7-day-old Arabidopsis and 8-day old maize leaves was detected at 514 nm excitation/522–564 nm emission, and imaged using the Zeiss LSM710 confocal microscope.

### Mapping-by-sequencing and bulked-segregant analysis

Two DNA bulks of 30 normal plants and 30 stomata-defective plants were obtained from the BC1F_2_ population derived from the *lsc-1* × B73 cross. The two pooled libraries were prepared and sequenced by Gene Denovo Biotechnology Company (Guangzhou, China). SNP-index and Δ(SNP) index values of two DNA pools, and the threshold of the significant SNPs (sSNPs)/total SNP ratio were calculated as described previously (Zhang and Panthee, 2020). Genes with non-synonymous SNPs within the candidate intervals were selected for analysis.

### Protein sequence alignment

The amino acid sequences of LSC and other homologous proteins were retrieved from NCBI (https://www.ncbi.nlm.nih.gov). Alignments of LSC (*Zm00001d045192*), ZmRNRL2(*Zm00001d036322*), OsRNRL1(*LOC_Os06g07210*), OsRNRL2(*LOC_Os02g56100*), SiSTL1(*Seita.4G058800*) and AtRNR1 (*At2g21790*), were conducted by CLUSTALW (Ebedes and Datta, 2004) and the output was edited by ESPript 3 (Robert and Gouet, 2014).

### dNTPs measurements

Leaves of 10-day-old wild type, *lsc-1*, *lsc-2*, OE-1 and OE-2 plants were harvested to measure dNTP (dATP, dTTP, dCTP, and dGTP) concentrations. The leaf samples were ground in liquid nitrogen until crushed, and ~ 0.2 g of crushed sample was weighed and placed in a 5 ml brown Eppendorf tube. To this, 1 ml of double distilled water was added, and fully homogenized. The extract was sonicated for 60 min and centrifuged at 1045 g for 10 min at 0–4 °C. The supernatant was then transferred into another Eppendorf tube, 1 ml of double distilled water was added to the remaining sediment, and processed once again following the above steps. The two supernatants were combined and mixed through a 0.22 μm filter. The obtained supernatant was stored in liquid nitrogen, and taken out and thawed before analysis. The measurements of dNTP concentrations were performed by Shanghai Sinobestbio Co., Ltd using a liquid chromatography–tandem mass spectroscopy (LC-MS/MS) system (SCIEX, USA), which was operated in multiple reaction monitoring mode.

### Generation of transgenic plants overexpressing *ZmLSC*

The 2445 bp CDS fragment of *ZmLSC* without a stop codon was amplified by PCR (Supplementary Table S1) and cloned into *p1300-VENUS*, a pCAMBIA1300 vector with a *VENUS* fragment inserted between the multiple cloning sites of S*ac*I and S*pe*I, and the construct of *pUBI::ZmLSC-VENUS* was generated using the restriction enzymes S*pe*I and DNA ligase, in which *ZmLSC* was driven by a ubiquitin promoter. Successful generation of all constructs was confirmed by DNA sequencing. Generation of the transgenic plants was performed by Edgene Biotechnology Co. Ltd. (Wuhan, China) using the maize inbred line B104 as the recipient. Basta herbicide (0.3% v/v) and PCR were used to screen for successful transgenic overexpression maize plants. For transgenic Arabidopsis plants, *pUBI::ZmLSC-VENUS* was introduced into the wild type Col-0 plants by the *Agrobacterium tumefaciens*-mediated floral dipping method (Clough and Bent, 1998).

### RNA extraction and qRT–PCR

Samples were taken from ~1.5 cm of the leaf base of 8-day-old maize seedlings for qRT–PCR analysis. Total RNA was isolated using the TRIzol reagent (Invitrogen Technologies, USA) according to the manufacturer’s protocol. Reverse transcription into cDNA was done with 5 μg total RNA in 20 μl reverse transcription mixture, using M-MLV Reverse Transcriptase (2641A, Takara, Japan). The cDNA was diluted to 200 μl, and 1 μl was used as the template for quantitative RT–PCR analysis. The relative expression of genes was detected by Roche light cycler480II Real-Time PCR system using the SYBR Green Master Mix (Vazyme Biotech Co. Ltd, Nanjing, China), according to the manufacturer’s instructions. Gene expression levels were calculated using the 2^–ΔΔCt^ method with *ZmActin1* (*Zm00001d010159*) and *ZmUbiquitin2* (*Zm00001d053838*) as the endogenous control genes. Quantitation was performed using at least three independent biological replicates. The primers used for RT–qPCR are listed in Supplementary Table S1.

## Results

### Identification and characterization of *lsc-1* mutant

**Fig. 1.**
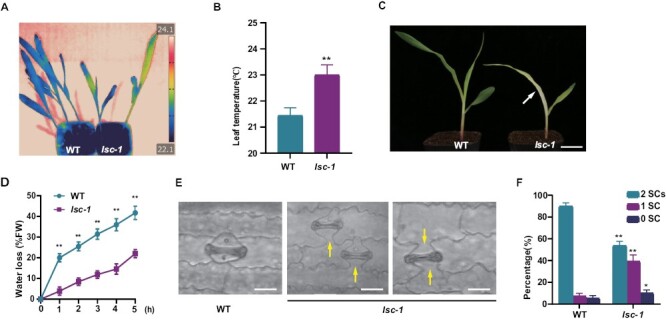
Phenotypic characterization of *lsc-1* mutant. (A) False-colour infrared image of 8-day-old seedlings of wild type (WT) and *lsc-1* mutant grown in soil. The temperatures (°C) of the first and second leaf surfaces are pseudo colour-coded according to the scale (inset). (B) Quantitative temperature measurements of wild-type and *lsc-1* leaves (*n*=10). (C) Phenotype of 8-day-old *lsc-1* and wild-type plants grown in soil. The *lsc-1* mutant shows growth retardation and is smaller than wild type. The arrowhead indicates the pale and bleached leaf in the *lsc-1* mutant. Scale bars=5 cm. (D) Transpirational water loss in *lsc-1* mutant and wild type. Detached leaves of 8-day-old seedlings were placed at 25 °C for 5 h and water loss was recorded at each hour. *n*=15 for each timepoint of each genotype. (E) The stomata containing only a single subsidiary cell or no subsidiary cell in the *lsc-1* mutant. A normal stoma in the wild-type leaf is shown in the left panel. Yellow arrowheads indicate defective subsidiary cells. These photographs were taken from the leaf base of the third leaf of 8-day-old seedlings grown in a growth chamber. Scale bars=25 μm. (F) Stomata (%) having different numbers of subsidiary cells at the base of the third leaf in the wild type and *lsc-1* mutant; 2 SCs, stomata with two subsidiary cells; 1 SC, stomata with only one subsidiary cell; 0 SC, stomata without subsidiary cell. Error bars indicate SD. *P<0.05, **P<0.01, Student’s *t*-test.

To identify novel regulators of stomata development in maize, we screened an ethyl methanesulfonate-generated mutant library in the B73 inbred background (Lu *et al*., 2018), and isolated a mutant, named *lsc-1*, based on the phenotype of leaf surface temperature and stomata morphology. The *lsc-1* mutant constitutively showed leaf temperatures ~1.5 °C higher than the wild type (B73), analysed by a far-infrared thermal imaging approach (Merlot *et al.*, 2002; Gao *et al.*, 2014; Fig. 1A, B). After germination, the cotyledon of *lsc-1* appeared normal, whereas the young newly grown leaves of *lsc-1* mutant were pale and bleached, especially at the leaf base (Fig. 1C). These bleached leaves always turned green slowly when they continued to expand, although the yellow stripe remained (Supplementary Fig. S1C). Compared with the wild-type plants, the *lsc-1* mutants were smaller and dwarf throughout the whole life cycle, and did not produce tassels (Supplementary Fig. S1A, B, D).

The leaf temperature change suggests that the *lsc-1* mutant may not be able to appropriately modulate water evaporation from its leaf surface. We measured the water loss rate and found that the water loss from leaves of the *lsc-1* mutant was significantly slower (*P*<0.01) than that of the wild type (Fig. 1D). As stomata are key for gas and water vapour exchange with the environment, we examined stomata of the young leaves of 8-day old seedlings of *lsc-1* and wild type maize plants. In wild type, the four-cell stomata complex comprises of two dumbbell-shaped GCs adjacent to two SCs. However, in the yellow-striped leaves of the *lsc-1* mutant ~38% of the stomata complex has only a single mature SC accompanying the normal GC; while at the side without SC divided from SMC, a triangular bulge of cell wall was formed, probably due to the stretching of GCs (Fig. 1E, F). A small proportion (~8%) of stomata with depletion of the two SCs can also be observed in *lsc-1* (Fig. 1E, F). Compared with the wild type, the *lsc-1* mutant had a higher proportion of stomata with only one SC or without SC, but lower proportion of normal stomata with two SCs (Fig. 1F). These observations indicated that both the formation and physiological function of stomata were defective in the *lsc-1* mutant. Nevertheless, the GC division appears normal, with two cells produced (Fig. 1E). Moreover, the stomatal density and aperture did not show significant differences (*P*>0.05) between wild type and *lsc-1* mutant (Supplementary Fig. S1E-H).

### SMC polarized division is defective in *lsc-1* mutant

**Fig. 2.**
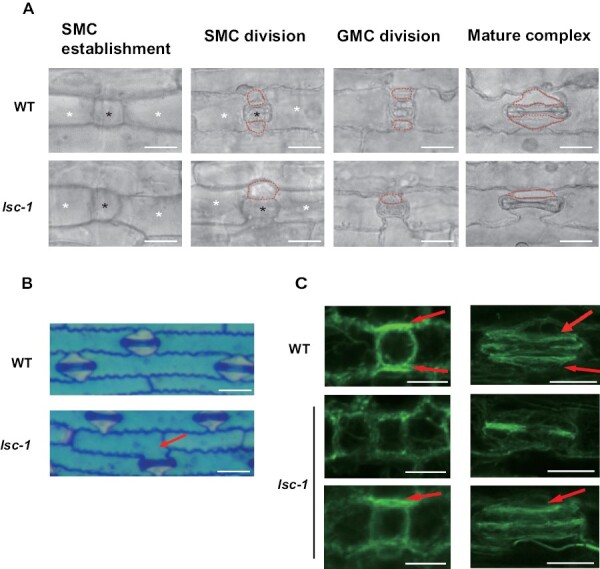
*LSC* is required for subsidiary cell (SC) development. (A) Stomatal development file of the base of the third leaf. Wild-type plants undergo normal SMC asymmetric division, resulting in two young SCs flanking the GMC, and they eventually develop into a complete stomata complex. In the *lsc-1* mutant, PDC can develop into GMCs at the early stages of this process. However, the SMCs undergo asymmetric division to form SCs at only one side (38%), but the longitudinal symmetric division of GMCs is not disturbed. Black asterisks indicate GMC; white asterisks indicate PDC; red dot lines indicate SC. Scale bars=50 μm. (B) Toluidine blue–stained epidermal peel from the third leaf of 8-day-old wild type (upper panel) and the *lsc-1* mutant. Guard cells (GCs) stain dark blue; SCs stain pink. Red arrowheads indicate defective SC formation in *lsc-1*. Scale bars=50 μm. (C) Confocal images of F-actin enrichment stained with AlexaFluor 488-phalloidin in wild type and the *lsc-1* mutant. In wild-type leaves, F-actin enrichment is observed at two sides of the SMC/GMC interfaces (red arrowheads). In the *lsc-1* mutants, no enrichment of F-actin patches was observed at the sides where SCs were lost. Left panels, early stage before SMC division. Right panels, late stage after SMC division. Scale bars=25 μm.

The SC loss in the *lsc-1* mutant prompted us to further examine the early development process of GCs and SCs. In wild-type plants, stomatal complexes in maize develop through an invariant sequence of coordinated asymmetric cell divisions (Vatén and Bergmann, 2012). After an asymmetric division that forms a GMC, its two lateral neighbour SMCs polarize toward the GMC, and subsequently form SCs flanking the GMC (Fig. 2A), then the GMC divides longitudinally to form a GC pair (Fig. 2A). However, in the *lsc-1* mutant with a single SC, only one SMC adjacent to GMC divides asymmetrically, and no division occurs in the other side (Fig. 2A). Toluidine blue staining confirmed the loss of SC in the *lsc-1* mutant, while normal SCs turned light pink (Fig. 2B). The subsequent symmetrical division of GMC into two GCs proceeded normally, suggesting that the SC differentiation defect is the dominant effect in stomata of the *lsc-1* mutant.

In the wild type, the initiation of SMC division needs its nucleus to be pulled to GMCs. During this process, a dense patch of F-actin accumulating at the SMC/GMC contact interface is required for SMC polarization, which presumably mediates the nuclear migration or anchor during cell division (Galatis and Apostolakos, 2004). In *lsc-1*, however, the defective early stomata showed F-actin enrichment at only one side of the GMC, or complete depletion of enrichment, which hints at a lack of the following SC differentiation step (Fig. 2C).

### Cloning of *LSC* gene

In order to characterize *LSC*, we back-crossed the *lsc-1* mutant (in the B73 background) to the inbred line B73, thereby generating BC1F_1_ plants and BC1F_2_ progenies. All F_1_ plants showed normal stomata phenotype as wild type. The F_2_ population consisting of ~120 individuals segregated in a 3:1 ratio of *lsc-1*-like mutant (30) and wild-type (94) plants (χ^2^ = 0.02174<χ^2^_0.05_ = 3.84), suggesting that *lsc-1* harboured a single recessive mutation that resulted in the defects in stomata.

**Fig. 3.**
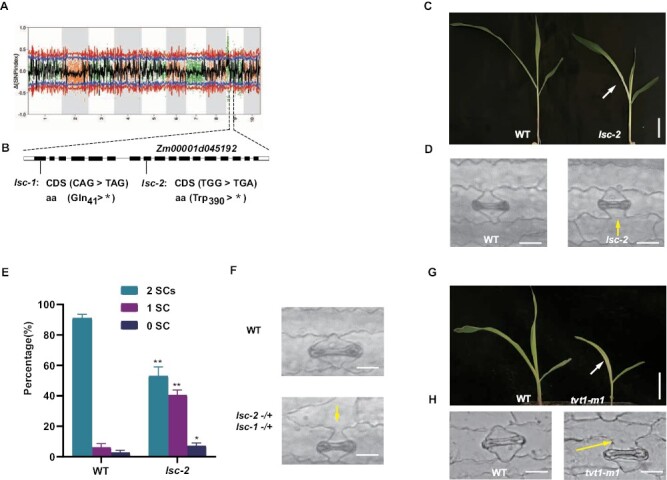
Identification of the *lsc-1* locus. (A) The *lsc-1* locus was identified by mapping-by-sequencing and was mapped to the short arm of chromosome 9 based on the Δ(SNP index). (B) A diagram showing the *Zm00001d045192* gene structure and the mutations. Boxes represent exons (black boxes indicate translated regions and white boxes indicate untranslated regions), and lines represent introns. The SNPs in *Zm00001d045192* were confirmed by re-sequencing. In *lsc-1*, this C-to-T point mutation in exon 1 leads to a premature stop codon, resulting in a predicted truncated peptide with 41 amino acids. In *lsc-2*, the G-to-A transition in exon 8 of *Zm00001d045192* also produces a premature stop codon, which is predicted to result in a truncated peptide with 390 amino acids. (C) Phenotype of 8-day-old seedling of *lsc-2,* showing a growth retardation phenotype like *lsc-1*. The arrowhead indicates the pale and bleached leaf. Scale bar=5.0 cm. (D) Aberrant stomata lacking subsidiary cells (SCs) in the *lsc-2* mutant, similar to that seen in *lsc-1*. (E) Percentage of stomata having different numbers of SCs at the leaf base of third leaves of 8-day-old wild-type and *lsc-2* mutants; 2 SCs, stomata with two subsidiary cells; 1 SC, stomata with only one subsidiary cell; 0 SC, stomata without subsidiary cell. (F) *lsc-1* and *lsc-2* are allelic. The heterozygous *lsc-1*(–/+) *lsc-2*(–/+) displays the same abnormal stomatal development as *lsc-1* and *lsc-2* with SC loss. Scale bars=50 μm. (G) The 8-day-old seedling of *tvt1-m1* shows a growth retardation phenotype like *lsc-1*. The arrowhead indicates the pale and bleached leaf. Scale bar=5.0 cm. (H) Aberrant stomata lacking SCs in the *tvt1-m1* mutant, similar to that seen in *lsc-1* and *lsc-2*. Yellow arrows indicate defective SCs. Scale bars=50 μm. WT, wild type. Error bars indicate SD. *P < 0.05, ***P < 0.001, Student’s *t*-test.

To identify the causal gene of *lsc-1*, we performed bulked segregant analysis (BSA)-seq by resequencing two DNA pools obtained from 30 mutant and 30 wild-type plants from the F_2_ population. The SNP index and Δ (SNP index) were calculated and used to perform association analysis. The mapping-by-sequencing via MutMap and subsequent genetic linkage analysis using the above population identified a candidate with C to T replacement in the first exon of *Zm00001d045192* at Chromosome 9 (Fig. 3A; Supplementary Fig. S2). This non-synonymous mutation generates a premature stop codon, resulting in a predicted truncated protein with 40 amino acids remaining (Fig. 3B). To confirm that the *lsc-1* mutant phenotype was caused by this mutation in *Zm00001d045192*, we obtained another maize mutant (http://www.elabcaas.cn/memd/), named *lsc-2*, with also a stop-gain mutation at exon 8 in this gene (Fig. 3B; Supplementary Fig. S2). The *lsc-2* mutant showed identical phenotypes as *lsc-1*, including dwarf plant, yellow-striped leaves, and aberrant stomata with one SC or without SC (Fig. 3C, D; Supplementary Fig. S3). In *lsc-2*, the proportions of stomata containing only one SC or no SC were both higher than that in wild-type plants. (Fig. 3E). Moreover, we crossed heterozygous *lsc-1* and *lsc-2* and found that the one fourth of *lsc-1 -/+ lsc-2 -/+* plants in F_1_ displayed the same abnormal stomata phenotype of percentage of SC loss in the leaves (Fig. 3F), indicating that *lsc-1* and *lsc-2* are allelic. To further confirm *Zm00001d045192* is the candidate gene, we analysed the *tvt1-m1* mutant, another null allele of *Zm00001d045192* containing a *Mu1.4* insertion in the third exon (Xie *et al.*, 2020). Apart from dwarf plants and pale leaves, we found that the *tvt1-m1* mutant also exhibited similar defective SC formation (Fig. 3G, H). Taken together, these results confirm that the null mutation of *Zm00001d045192* is the molecular basis for the *lsc* mutant phenotype.

### LSC is involved in dNTP production

The gene *LSC* (*Zm00001d045192*) encodes a large subunit of RNR with 815 amino acid residues attributed to the production of dNTPs for DNA synthesis and repair (Xie *et al.*, 2020). Its homologs are present in almost all living organisms, including a paralog in the maize genome. Protein sequence alignments showed that LSC shared 97%, 96%, 93%, 89%, and 86% sequence identity with ZmRNRL2, SiSTL1(Tang *et al.*, 2019), OsRNRL1, OsRNRL2 (Yoo *et al.*, 2009) and AtRNR1, respectively (Supplementary Fig. S4) (Garton *et al.*, 2007; Tang *et al.*, 2012).

**Fig. 4.**
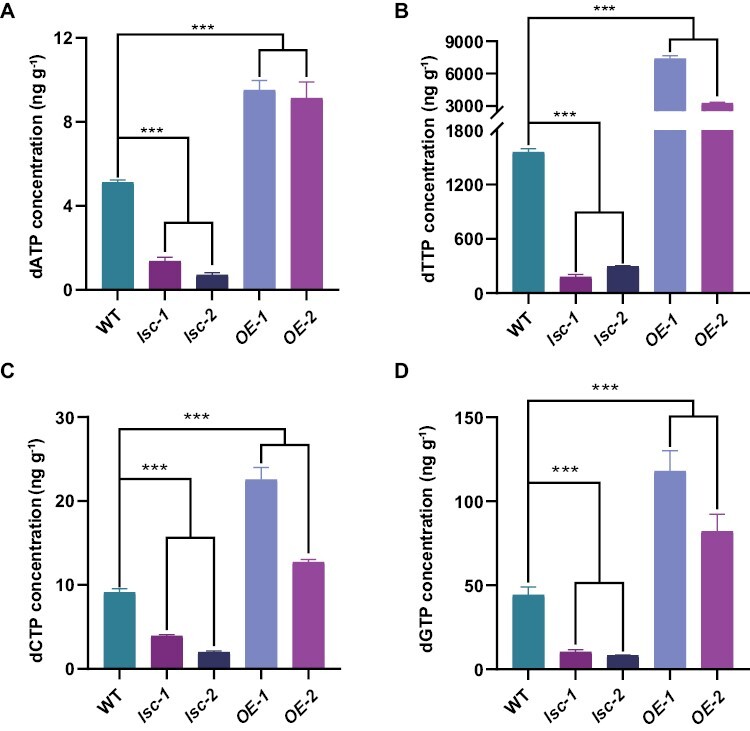
LSC regulates dNTP synthesis. (A) dATP concentrations in wild type, *lsc-1*, *lsc-2*, *OE-1* and *OE-2* leaves. (B) dTTP concentrations in wild-type, *lsc-1*, *lsc-2*, *OE-1* and *OE-2* leaves. (C) dCTP concentrations in wild-type, *lsc-1*, *lsc-2*, *OE-1* and *OE-2* leaves. (D) dGTP concentrations in wild-type, *lsc-1*, *lsc-2*, *OE-1* and *OE-2* leaves. All samples are taken from the third leaf of 8-day-old seedlings grown in soil. Three independent samples for each genotype were examined, each value representing the means (± SD). One-way ANOVA was used to detect significant differences (***P < 0.001). WT, wild type.

We speculated that the loss of LSC may negatively affect the RNR activity and result in an insufficient and/or unbalanced supply of dNTPs in the mutant cells during early development. To examine this possibility, we measured the dNTP concentrations in the leaves of *lsc-1*and *lsc-2* mutants by LC-MS. All the four nucleotides (dATP, dTTP, dGTP, and dCTP), especially dTTP, the most abundant in living tissues, were significantly decreased (*P*<0.001) in the *lsc-1* and *lsc-2* mutants, compared with the wild type (Fig. 4A-D). This strongly suggests that the *lsc-1* and *lsc-2* alleles are negative mutations for dNTP biosynthesis in terms of RNR activity.

### LSC affects the expression of genes associated with cell cycle progression and stomata development

**Fig. 5.**
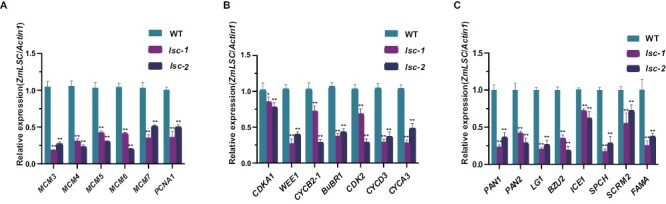
Expression analysis of genes associated with DNA replication, cell cycle and stomatal development in the wild-type and *lsc* mutants. (A) qRT–PCR analysis of the expression of genes associated with DNA replication, *minichromosome maintenance* (*MCM3*/*4*/*5*/*6*/*7*) and *proliferating cell nuclear antigen1* (*PCNA1*). (B) qRT–PCR analysis of the expression of genes involved in cell cycle progression, *cyclin-dependent kinase1*(*CDKA1*), *WEE1*, *cyclin B2-1*(*CYCB2-1*), *benzimidazoles related 1*(*BuBR1*), *Cyclin-dependent kinase 2*(*CDK2*), *cyclin D3* (*CYCD3*) and *cyclin A3*(*CYCA3*). (C) qRT–PCR analysis of the expression of genes affecting stomatal development, *pangloss1* (*PAN1*), *pangloss2* (*PAN2*), *Liguleless1* (*LG1*), *bizui2* (*BZU2*), *INDUCER OF CBF EXPRESSION1* (*ICE1*), *SPEECHLESS* (*SPCH*), *SCREAM2* (*SCRM2*) and *FAMA*. *ZmActin1* was used as an internal control; *n*=3 for each gene. Values are means ±SD. WT, wild type.

Insufficient dNTP synthesis due to the dysfunction of the RNR enzyme is known to influence DNA replication and repair, which lead to impeded cell cycle progression and ultimately cell division (Reichard, 1988; Nordlund and Reichard, 2006). To test whether LSC affects DNA synthesis and cell division, we performed quantitative RT–PCR to compare the expression levels of relevant genes involved in DNA replication and cell cycle reported previously using wild type and *lsc* mutant leaves (Menges *et al.*, 2005; Juraniec *et al.*, 2016). The results showed that the genes we examined were all down-regulated in both *lsc-1* and *lsc-2* mutants (Fig. 5A, B; Supplementary Fig. S5A, B), which supported our speculation that less SC number and retarded plant growth in *lsc-1* and *lsc-2* mutants might be the result of limited dNTP supply for cell division. Furthermore, we analysed the important genes *FAMA*, *PANGLOSS* (*PAN1*), *PAN2*, *BIZUI2* (*BZU2*), *SPEECHLESS* (*SPCH*)*, ICE1,* and *SCRM2* that are known to be involved in stomatal development. Their transcript levels were also significantly lower (*P*<0.01) in both *lsc-1* and *lsc-2* mutants (Fig. 5C; Supplementary Fig. S5C), consistent with the absent SC phenotype.

As chloroplast development has been reported to be affected by insufficient dNTPs in other RNR dysfunction mutants (Niu *et al.*, 2017; Tang *et al*., 2019), we examined the chlorotic leaves of *lsc-1* mutant and found that the chloroplast number was reduced (Supplementary Fig. S6A, B). This further confirmed the disrupted RNR activity in the *lsc-1* mutant.

### Overexpression of *LSC* promotes vegetative growth

**Fig. 6.**
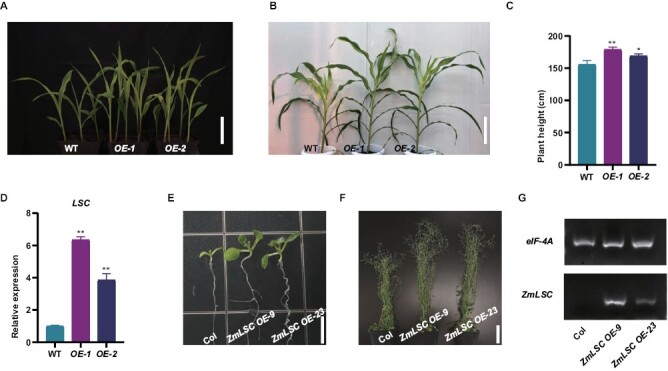
Overexpression of *LSC* promotes vegetative growth in maize and Arabidopsis. (A) Phenotype of seedlings of 8-day-old wild-type and *LSC* overexpression lines (*OE-1* and *OE-2*) in B104 genetic background grown in soil. The overexpression lines grow faster and are taller than wild type. Scale bar=5 cm. (B) Phenotype of 50-day-old *OE-1*, *OE-2*, and wild-type plants grown in the greenhouse under 16 h light/8 h dark at 28–30 °C. Scale bar=20 cm. (C) Quantification of the plant heights of WT, *OE-1* and *OE-2* at the tasselling stage; *n*=10. (D) *LSC* expression in 8-day-old maize seedlings of WT, *OE-1*, and *OE-2* . (E) Transgenic Arabidopsis plants overexpressing *ZmLSC.* Two different OE lines are shown, *ZmLSC OE-9* and *ZmLSC OE-23*. The 7-day-old seedlings of overexpression lines displayed bigger cotyledons than wild type (Col-0). Scale bar=0.5 cm. (F) The 60-day-old Arabidopsis plants overexpressing *ZmLSC* are taller than the wild-type (Col-0) plants. Scale bar=5 cm. (G) Validation of *ZmLSC* expression in transgenic Arabidopsis plants using RT–PCR; the PCR products analysed via DNA electrophoresis are shown. *AteIF-4A* was used as the internal control. Error bars indicate SD. *P<0.05, **P<0.01, Student’s *t*-test.

To further validate the function of *LSC*, we generated overexpression (OE) lines in maize B73 background with *pUBI::LSC-VENUS* . Two independent positive transgenic lines (OE-1 and OE-2) were used for investigation. Fluorescence observation in leaf epidermal cells (Supplementary Fig. S7A) and qRT–PCR indicated the overexpression of *LSC* (Fig. 6D; Supplementary Fig. S5D). The OE plants grew faster, and not only the seedlings, but the adult plants were also taller than the wild type B104 (Fig. 6A-C). However, we did not observe any changes in the morphology and density of stomata in these OE lines (Supplementary Fig. S8A-D). We further analysed the concentration of dNTPs in these OE plants and found all four nucleotide molecules were increased to varying degrees compared with that in wild-type plants (Fig. 4A-D). These results suggest that enhanced *LSC* expression would lead to overproduction of dNTPs, and ultimately promote tissue growth, but not stomata formation.

In addition, we also generated transgenic Arabidopsis plants using the same OE construct, with the fluorescence in leaf epidermal cells as a selection marker of positive lines (Supplementary Fig. S7B).We found that the ectopic expression of maize *LSC* in Arabidopsis behaved similar to that in maize, because it also promoted growth, which was observed as enlarged seedlings and taller plants (Fig 6E-G). These results may suggest a possible conserved RNR functional pathway in maize and Arabidopsis.

## Discussion

### *lsc-1* and *lsc-2* are novel mutant alleles of maize *RNRL1*

In our study, we identified a maize mutant *lsc-1* displaying pleiotropic phenotypes, including dwarf infertile plants, chlorotic leaves, aberrant stomata, and compromised chloroplast development. Gene cloning showed that *LSC* encodes a large unit of RNR (Fig. 3A) that is responsible for dNDP and dNTP synthesis in both animals and plants (Jordan and Reichard, 1998). Although the phenotypes of infertile, chlorotic leaves, and chloroplast have been reported in several RNR mutants of maize, rice, and Arabidopsis (Wang and Liu, 2006; Chen *et al.*, 2015; Xie *et al.*, 2020), SC absence was not observed in them. It has been previously reported that two alleles of *Zm00001d045192*, named *tvt1-R* and *tvt1-m1*. *tvt1-R* contain an amino acid change from Arg to His, exhibiting dwarf, striped leaves and no tassels at only restrictive high temperature; whereas *tvt1-m1*, a frame-shift allele with a Mu insertion, has striped leaves and no tassels at both permissive and restrictive temperatures (Xie *et al.*, 2020). Our analysis showed that *lsc-1*, *lsc-2*, and *tvt1-*m1 have not only the similar leaf and tassel phenotypes, but also the same SC defect under normal growth conditions (Figs 1C, E, 3C, D, G, H). We therefore confirmed that *Zm00001d045192* is the candidate gene responsible for the SC phenotype, and *lsc-1* and *lsc-2* are strong null alleles as they are both premature stop-gain mutants.

SCs are important partners of GCs in monocots, supporting the opening and closure of stomata when exposed to stimuli, and participating in the control of gas exchange during photosynthesis and water vapor loss due to transpiration. Therefore, the reduced SCs may weaken the movement of GCs, and finally restrain photosynthetic activity due to less income of CO_2_ and solute flow in vascular tissues because of reduced transpiration pull. These impacts might also result in the inhibition of plant growth. We hypothesize that the chloroplast deficiency may also suppress photosynthesis in the *lsc* mutants.

### SC development might be sensitive to dNTP deficiency

The dNTP concentration fluctuates during different stages of the eukaryotic cell cycle because of changes in the activity of RNR (Chabes and Stillman, 2007; Tang *et al*., 2019). Dynamic dNTP levels are required for the normal replication of DNA and the promotion of cell cycle progression and cell division (Abid *et al.*, 1999; Chabes and Stillman, 2007). The *lsc-1* and *lsc-2* mutants have lower concentrations of four dNTPs (Fig. 4A-D) due to the loss of function of a large subunit of RNR. Moreover, the reduced expression of genes involved in DNA replication and cell cycle regulation (Fig. 5A-C) supported the defect of cell cycle progression in *lsc* mutants.

In the study of *rnr* mutants, Garton *et al.* (2007) suggested that under a limited dNTP supply, the inhibition of chloroplast DNA replication may be a primary cause of the aberrant development. Based on the study of *virescent3* and *stripe1* mutants in rice, it was speculated that the dNTP deficiency led to blocked plastid DNA synthesis first, rather than nuclear DNA replication. Thus, it was proposed that the nuclear genome is critical for cell maintenance and division in higher plants, while the plasmid genome is relatively less important for cell survival (Mahapatra *et al.*, 2021). This sacrifice mechanism might be a smart way to maintain the growth and development of plants. In the *lsc* mutants, not only chloroplast development but SC development was also compromised during the early stages of leaf growth. It is thus speculated that the abnormal SC formation may potentially represent a cell autonomous defect relying on the collapse of a primary metabolism pathway. It was also noted that abnormal stomata lacking one SC were in greater number than that lacking two SCs (Figs 1F, 3E). A hypothesis that lacks further evidence at present is that the SC sacrifice might be another mechanism to save limited dNTPs at stages with a high amount of cell division events, to maintain other essential division and growth. The normal germination and cotyledon development are likely to be a result of the dNTP supply from the storage tissues at that early growth stage (Shewry, 2001; Crozier *et al.*, 2006).

The requirement of dNTPs varies during different cell cycle stages. Thus, an insufficient or constitutive supply of dNTPs can disrupt the normal cell cycle and division (Tang *et al*., 2019). It has been reported that *Tvt1* overexpression plants which showed very high expression levels of *Tvt1* exhibited similar yellow-striped leaves as the *tvt1* knockout mutants (Xie *et al.*, 2020). This is supposed to be the result of very high expression levels, leading to the co-suppression of gene transcripts (Xie *et al.*, 2020). Our overexpression lines in this study did not show extremely high levels of gene expression and striped-leaf phenotype, but promoted plant growth in both maize and Arabidopsis (Fig. 6A, B, E, F), which may provide a transgenic resource for crop improvement.

## Supplementary data

The following supplementary data are available at *JXB* online.

Fig. S1. Plant and stomatal phenotype of the wild type and *lsc-1* mutants.

Fig. S2. Mutation validation by DNA sequencing.

Fig. S3. Plant phenotype of the wild type and *lsc-2* mutant.

Fig. S4. The amino acid sequence alignment of LSC and homologs.

Fig. S5. Quantitative real-time PCR using a second endogenous control gene.

Fig. S6. Chloroplast development in the wild type and *lsc-1* mutant.

Fig. S7. Fluorescence observation in the leaf cells of transgenic maize and Arabidopsis plants.

Fig. S8. Stomatal phenotype in the wild type and *LSC* overexpression plants.

Table S1. Primers used in our study.

erad153_suppl_Supplementary_Figures_S1-S8_Table_S1

## Data Availability

All data supporting the findings of this study are available within the manuscript and its supplementary data published online.
