# Supplementary material for: Maize LOST SUBSIDIARY CELL encoding a large subunit of ribonucleotide reductase is required for subsidiary cell development and plant growth
Source: J Exp Bot. 2023 Apr 27;74(15):4449–60. doi: 10.1093/jxb/erad153 (PMC10433938; doi:10.1093/jxb/erad153)
Supplement: erad153_suppl_Supplementary_Figures_S1-S8_Table_S1 [file erad153_suppl_supplementary_figures_s1-s8_table_s1.pdf]

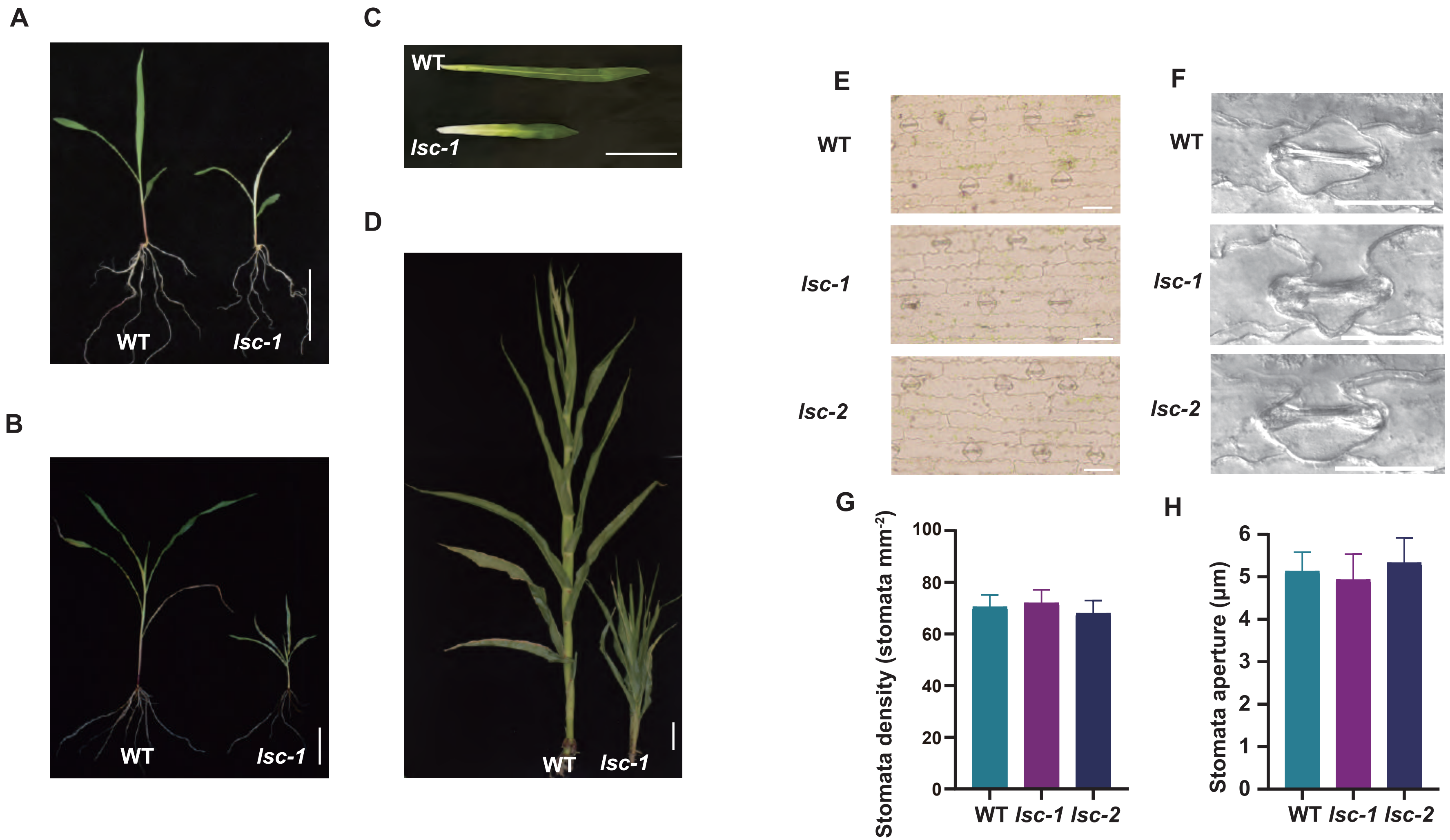

Supplementary Figure S1. Plant phenotype of WT and *lsc-1* mutant.
(A) 8-day-old wild-type and *lsc-1* plants in the B73 genetic background grown in a growth chamber (16 h/8 h light/dark cycle at 26–30˚C). Scale bars, 10 cm. (B) 25-day-old wild-type and *lsc-1* plants in the B73 genetic background grown in a growth chamber (16 h/8 h light/dark cycle at 26–30˚C). Scale bars, 10 cm. (C) Leaf phenotype of WT and *lsc-1* mutant. Picture were taken from the third leaf of 8-day-old seedlings. Scale bar, 5cm. (D) Wild-type and *lsc-1* plants in the B73 genetic background at reproductive growth stage, grown in greenhouse under 16 h light/8 h dark at 28–30 °C. Scale bars, 10 cm. (E) and (G) Stomatal density in WT, *lsc-1* and *lsc-2.* n=10, Error bars indicating SD, Student's *t*-test.Scale bars, 100 µm.(F) and (H) Stomatal aperture in WT, *lsc-1* and *lsc-2.* n=10, Error bars indicating SD, Student's *t*-test. Scale bars, 50 µm.

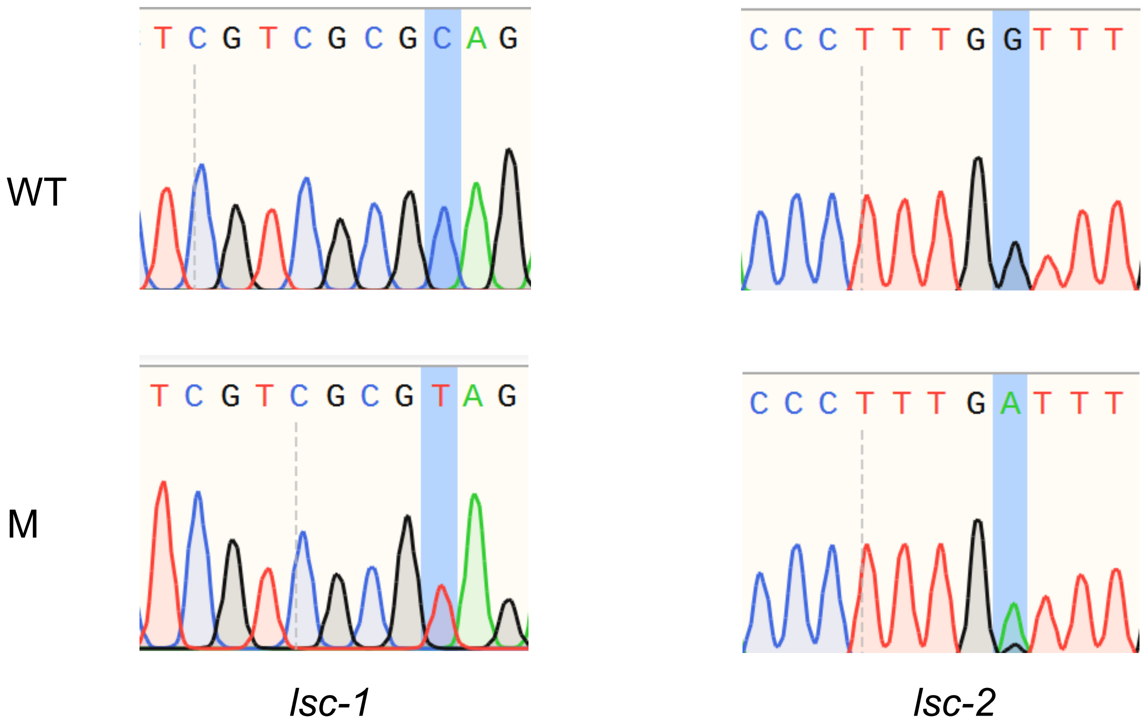

Figure S2. Mutation validation by DNA sequencing.
Re-sequencing results indicate the mutant nucleotides in wild type, *lsc-1* and *lsc-2* mutants.

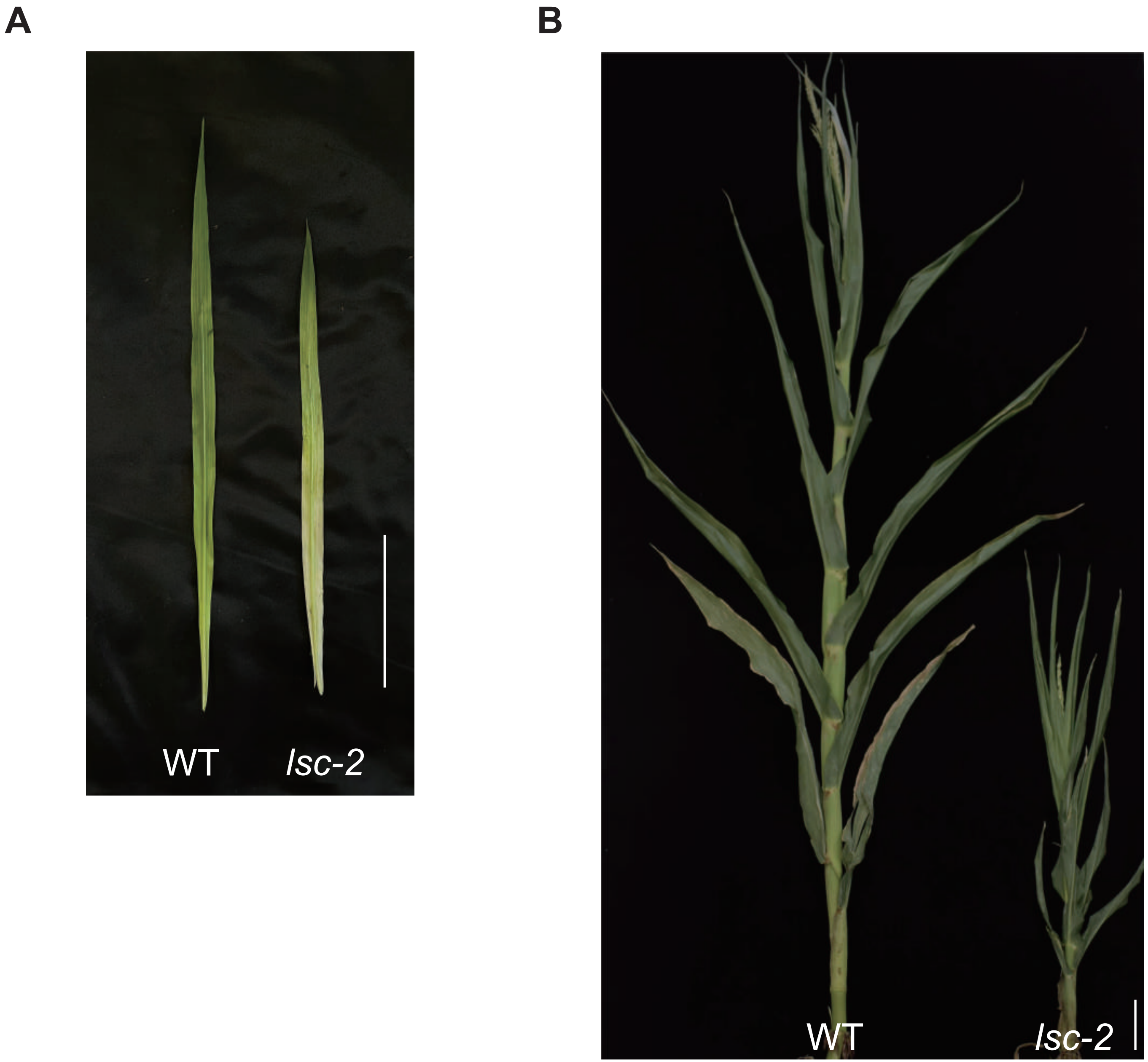

Figure S3. Plant phenotype of WT and *lsc-2* mutant

(A) leaves of 8-day-old seedlings of wild type and *lsc-2* mutant. Scale bar, 5 cm. (B) Wild type and *lsc-2* plants at reproductive growth stage grown in greenhouse. Scale bar, 20 cm.

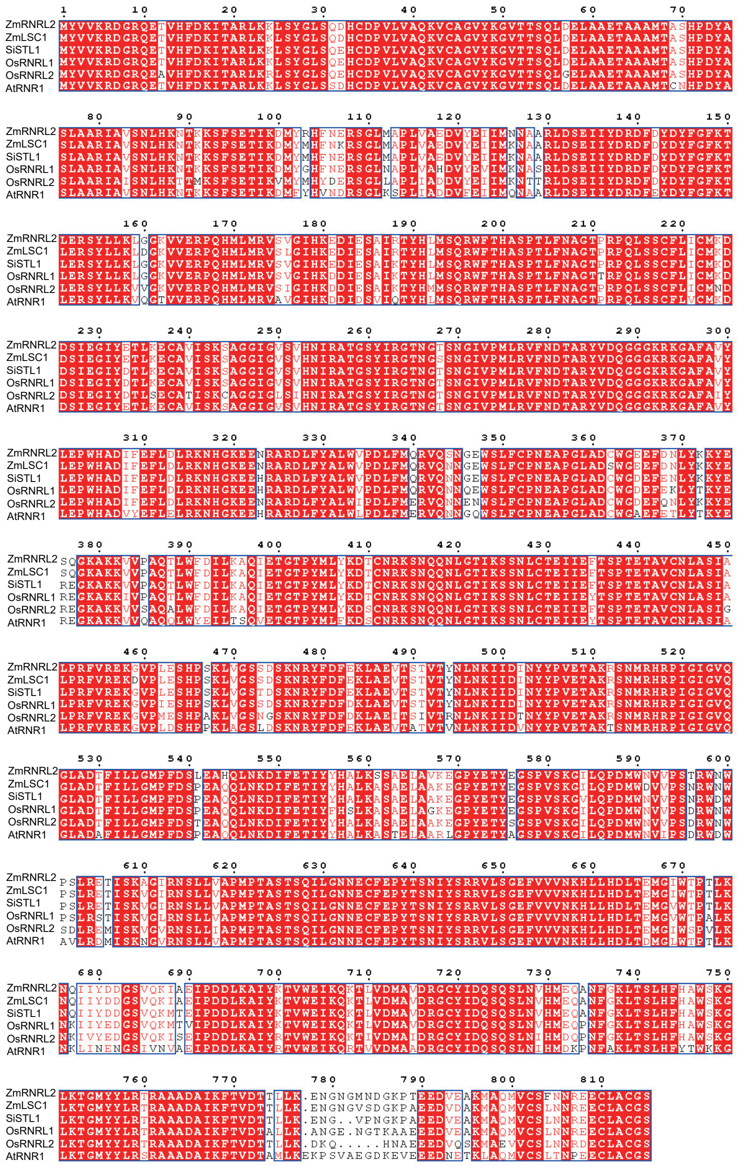

Figure S4. Sequence alignment of LSC and homologous.

The amino acid sequence of LSC was compared with its homologous in other species using CLUSTALW. Letters with the red background indicate identical amino acid residues, and pink letters indicate amino acid residues that are greater than 80% conserved. ZmRNRL2 (Zea mays, *Zm00001d036322*), ZmLSC (Zea mays, *Zm00001d045192*), OSRNRL1 (Oryza sativa, *LOC Os06g-07210*), OSRNRL2 (Oryza sativa, *LOC Os02g56100*), SiSTL1(Setaria italica, *Seita.4G058800*) and AtRNR1 (Arabidopsis thaliana, *At2g21790*)

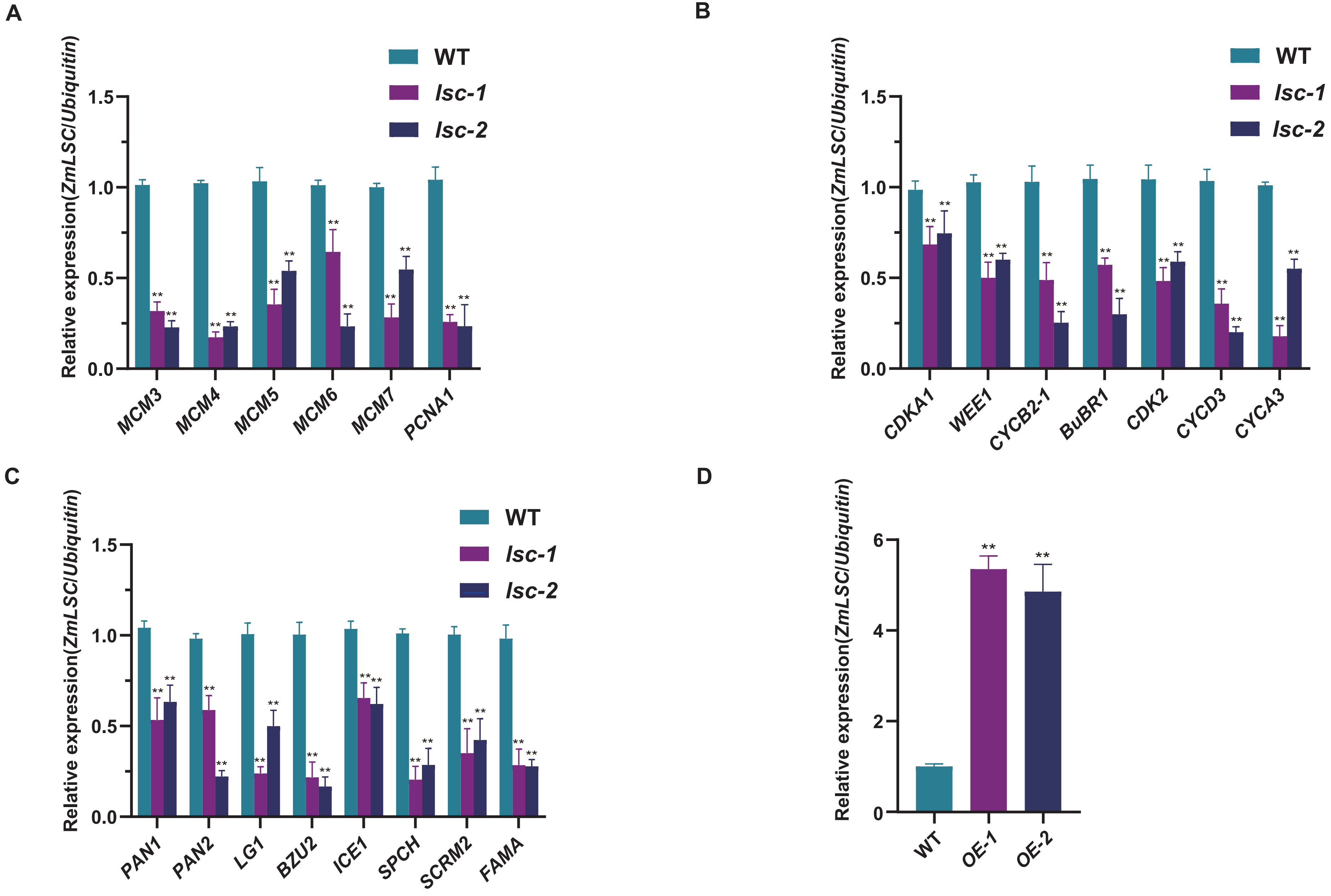

Supplementary Figure S5. Quantitative real-time PCR using a second endogenous control gene.

(A) The exprssion levels of *minichromosome maintenance*(*MCM3*/*4*/*5*/*6*/*7*) and *proliferating cell nuclear antigen1*(*PCNA1*) were determined by qRT-PCR. (B) The exprssion levels of *cyclin-dependent kinase1*(*CDKA1*), *WEE1*, *cyclin B2-1*(*CYCB2-1*), *benzimidazoles related 1*(*BuBR1*), *Cyclin-dependent kinase 2*(*CDK2*), *cyclin D3* (*CYCD3*) and *cyclin A3*(*CYCA3*) were determined by qRT-PCR. (C) The exprssion levels of *pangloss1*(*PAN1*), *pangloss2*(*PAN2*), *Liguleless1*(*LG1*), *bizui2*(*BZU2*), *INDUCER OF CBF EXPRESSION1*(*ICE1*), *SPEECHLESS*(*SPCH*), *SCREAM2*(*SCRM2*) and *FAMA* were determined by qRT-PCR. (D) *LSC* expression in WT, *OE-1* and *OE-2* maize plants at 8-day-old seedlings. *ZmUbiquitin2* was used as an internal control. Values are means ± SD of at least three biological replicates. WT, wild type. *$P < 0.05$, **$P<0.01$, Student's *t*-test.

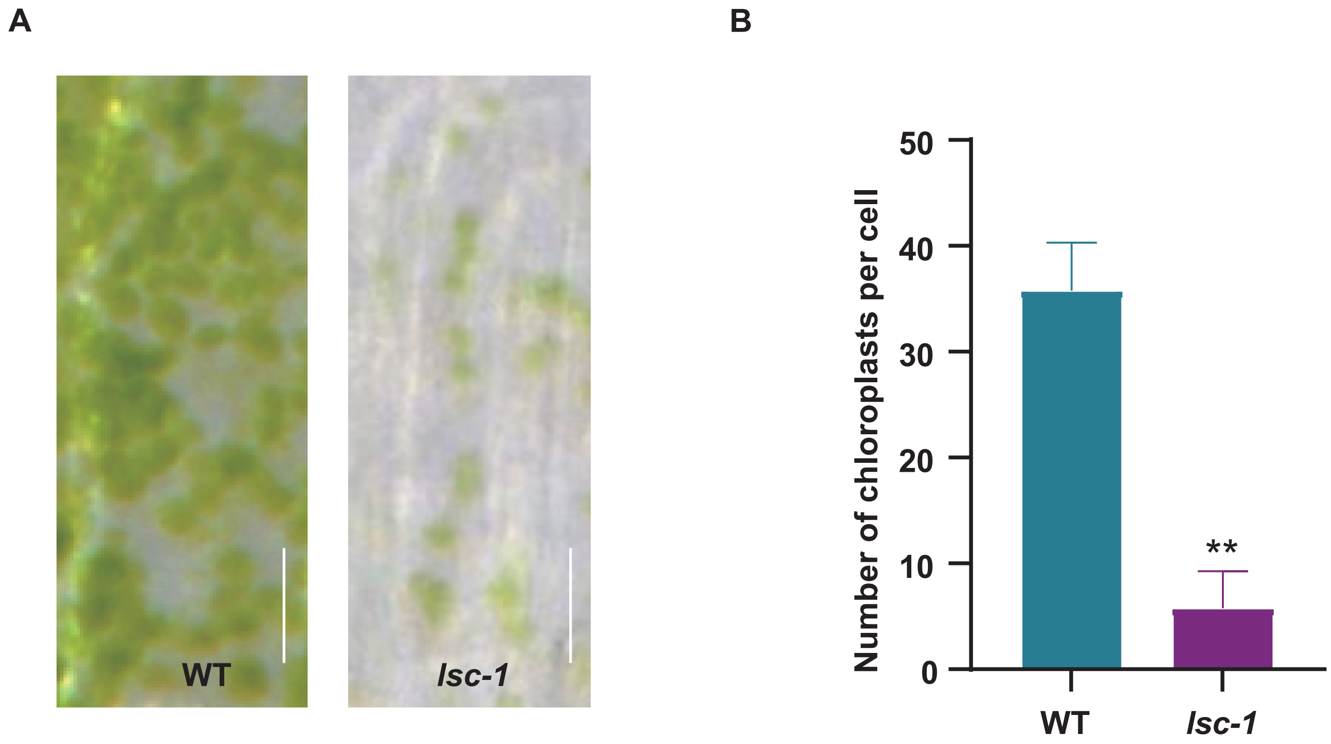

Supplementary Figure S6. Chloroplast development in wild type and *lsc-1* mutant.
(A) Microscopic observation of chloroplasts in the leaf base of the third leaf from 8-day-old wild type and *lsc-1* mutant. Scale bars, 5 μm. (B) Quantitative analysis of chloroplast numbers in the leaf cells of wild type and *lsc-1* mutant. Error bars, ±SD. n=15, ***P*<0.01, Student's *t*-test.

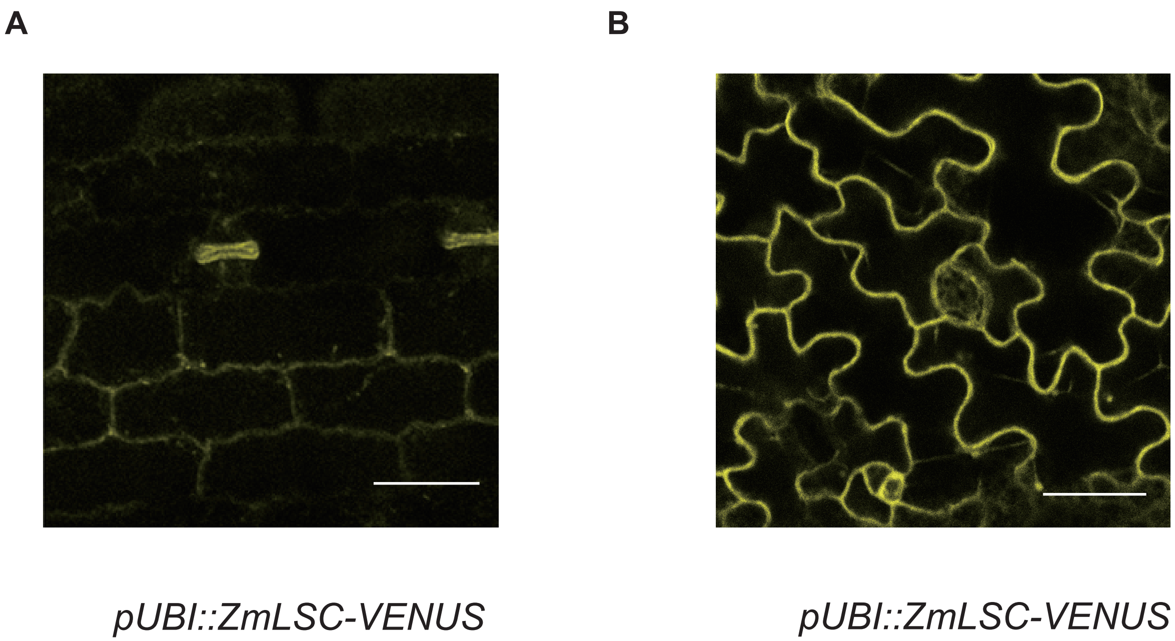

Supplementary Figure S7. Fluorescent observation in the leaf cells of transgenic of maize and *Arabidopsis* plants.
(A) Confocal image of the epidermal cells of transgenic maize plants expressing *pUBI::ZmLSC-VENUS*. (B) Confocal image of the epidermal cells of transgenic *Arabidopsis* plants expressing *pUBI::ZmLSC-VENUS*. Scale bars, 100 µm.

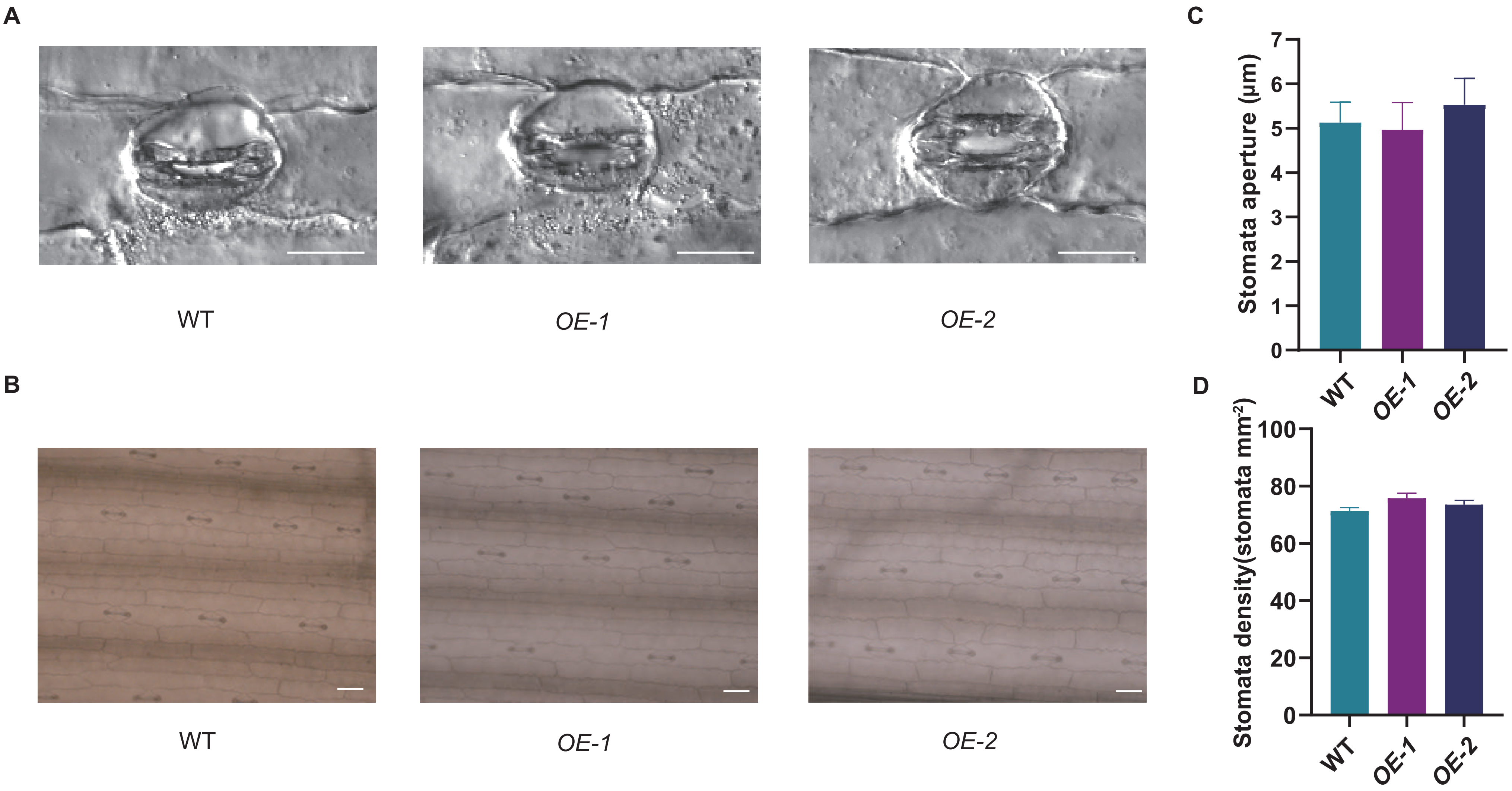

Supplementary Figure S8. Stomatal phenotype in the wild type and *LSC* overexpression plants.
(A) Confocal images show the stomata are normal in wild type and *LSC* overexpressed maize plants (*OE-1* and *OE-2*). Scale bars, 50 μm. (B) Images of leaf abaxial epidermal layers from 8-day-old seedlings of wild type and overexpression plants. Scale bars, 100 μm. (C) Stomata aperture in wild type and overexpression plants. (D) Stomata density in wild type and overexpression plants. No significant differences were detected between wild type and overexpression plants using the unpaired Student's *t*-test. n = 25. WT, wild type.

Table S1 Primers used in our study.

Name	Sequence F	Sequence R
A: Primers used in mutant site identification		
lsc-1	CTTCATTACTCCTCGCCTCTTCC	GAGGATTTCGGGGTGGGTTAC
lsc-2	GCCCTAATGAAGCTCCAGGG	TCAGTGCACAGGTTTGACGA
B: Primers used in vector construction		
pUBI::LSC/ZmRNRL1-VENUS	gcgcgccatttaaatactagtATGTACGTGGTCAAGAGGGACG	catggtggatcccatactagtGCTTCCACACGCCAGGCA
B: Primers used in gene expression examination		
AteIF4A- qRT	CGTGGTTTCAAGGACCAGAT	ATTGATCGCAACACCCTTTCT
ZmLSC- qRT	CTCAGTAACCAGAACGAAGTCT	CGATCCAATGCAAAGAGTGTAG
ZmActin1- qRT	TCACCCTGTGCTGCTGACCG	GAACCGTGTGGCTCACACCA
ZmUbiquitin2-qRT	TGGTTGTGGCTTCGTTGGTT	GCTGCAGAAGAGTTTTGGGTACA
ZmCDKA1-qRT	CTTCGAGTACCTGGATCTGGA	GCAACACCGTGGAGTATC
ZmWEE1-qRT	GACTTCCTGAGCCACAATCTATG	GTCGTCGTCGAACTCGTTG
ZmCyclinB2-1-qRT	AAGCTGCAGATGCAGATAAACAGC	GCTGGCAACGATTGATAGCACAC
ZmBUBR1-qRT	TCAAAGTGTGGCTGGAATACGC	ACTCCAGCAACGATGCGTAAGAC
ZmCDK2-qRT	CACGGCAACATCGTCAGGTTAC	TCTTAGCAAACTCCGGGCAAGAG
ZmCYCD3-qRT	ACTATTTGGACAAGGAGAGGGT	ACCTGAAGCGTACCACAACT
ZmCYCA3-qRT	ACACCCATCCCTGGAGCAAG	TGTATTTCTCCCGGATCGCC
ZmMCM3-qRT	GTGCCATGGTTCTTGCTGATCGTGGTGT	GGAAAGCAGTGAATCCGGAAGCCCGATA
ZmMCM4-qRT	GAGCTCGAGGTCTTGGACTTGCAGACAC	TGCTTCGCTGAGACGGATCAAACTCTCT
ZmMCM5-qRT	AAAATGAGGCAGCAAGCTCACGAGACAG	ATCTTGCAGCATCAACGGTGGAGACATT
ZmMCM6-qRT	ATGACCAAGCAGCGGATAATGG	ATGCCAGCTAAACCATCTCCATC
ZmMCM7-qRT	GCAGAGCACGTACCAAAGGGCCACATTC	GCAACTAATCCTGCGCGCATCGCTCTAA
ZmPCNA1- qRT	CCATCGTCCGCATGCCTTCTTCTGAGTT	CAAAGGTCAGGGAAACCGGCTCTTGCAT
ZmFAMA- qRT	CACCAACATCACCACCATC	GAAGCTGAGGATCTGGTGC
ZmPAN1- qRT	GTTCGGCGTCACATGCTCCA	CAGCTCCGACAGCAGCGAGA
ZmPAN2- qRT	GGCTTGCGTGGGTACTACTG	GGTCAGTGGAGGACCTCTTCTT
ZmLG1- qRT	AGATACCACCGTCGCCACAAG	ACGTATCCAGAGGAAAGCCAT
ZmBZU2- qRT	GCGCTCCCACCTCCTCTCCTCC	CGGAGATCTTGGCCTCGACGTC
ZmSPCH-qRT	CGAGTGAAGATGGTGCGGTA	TGGTCTCCTCGCTTGACGTA
ZmICE1-qRT	TTCAACCAAATTACCAAACCC	TTAATTTCTTCAGGCACCATTC
ZmSCRM2-qRT	ATCTTCAAGGCCGAGCTGTG	AGCGGTCTTGTTGTCCTAGC

Nucleotides indicating restriction sites are underlined.
